# Data of a sub-analysis of the DEMDATA study: characteristics of Austrian and Czech nursing homes residents

**DOI:** 10.1186/s13104-019-4495-6

**Published:** 2019-07-29

**Authors:** Margit Höfler, Paulina Ratajczak, Anna Beránková, Doris Prieschl, Michal Šteffl, Iva Holmerová, Stefanie R. Auer

**Affiliations:** 1 Danube University Krems, Dr. Karl-Dorrekstrasse 30, 3500 Krems, Austria; 2 Centre of Expertise in Longevity and Long-Term Care, Faculty of Humanities, Charles University, Prague, Czech Republic; 3 MAS Alzheimerhilfe, Lindau Strasse 28, 4820 Bad Ischl, Austria; 4 Faculty of Physical Education and Sport, Charles University, Prague, Czech Republic

**Keywords:** Dementia, Nursing homes, Epidemiological data, Austria, Czech Republic

## Abstract

**Objectives:**

The aim of this data paper is to provide the data set of a sub-analysis of the DEMDATA study data. In the DEMDATA study, epidemiological data on the prevalence and severity of dementia, as well as functioning, behavioral problems and other health related factors in residents living in Austrian and Czech nursing homes were collected. The DEMDATA project further provides information on relatives’ perception of the life Quality of residents, care team burden as well as environmental factors. Participating nursing homes were randomly drawn and stratified. Inclusion criteria for participation were that the resident was living permanently in the institution and that he/she and/or a legal representative (where relevant) had signed an informed consent.

**Data description:**

This paper provides data of cognitive, functional and behavioral assessments as well as other health related information of 1085 residents living in Austrian and Czech nursing homes. For each resident, several measurements on his or her cognitive, functional, and behavioral status are available. Also further health-related factors such as quality of life, pain, numbers of falls and hospital stays are provided.

## Objective

Comprehensive information about the epidemiology of the prevalence and severity of dementia, functioning and behavioral problems for residents in European nursing homes is essential in order to develop optimal care concepts. Often such data are collected indirectly via surveys and the methods of assessment vary across countries, making it difficult to compare findings. In order to overcome this deficit, we conducted the DEMDATA study that aimed to collect data on the cognitive and functional status, behavioral symptoms as well as other health-related factors in Austrian and Czech nursing homes. In addition, environmental factors and the situation of the relatives and the care team were assessed. We created a common study protocol which was applied in both countries [1]. In the current paper, we describe data collected during the evaluation of the residents. These data were recently published in order to compare the situation of the residents in both countries [2].

## Data description

The sample consists of 571 residents of eight Austrian and 514 residents of 14 Czech nursing homes [3, 4] (see Table 1). In the randomly selected nursing homes, all residents were invited to participate in the study. The only inclusion criterion was the given informed consent by the resident or a legal representative. Excluded from the study were residents with an acute serious health crisis or if they were in the process of dying. Sociodemographic data such as gender, age and country were removed from the data set because of data privacy. However, these data can be provided upon written request. The mean residents’ age was 84.4 years (SD = 8.33; Austria) and 84.6 years (SD = 7.51, Czech Republic) and the majority was female (AUT: 73.4%, CZ: 77.8%). On average, residents lived in the respective nursing home for 3.4 years (SD = 4.5) in Austria and 3.1 years (SD = 3.6) in the Czech Republic. The data collection was conducted between September 2016 and June 2017 (Austria) and between August 2016 and August 2017 (Czech Republic) and performed by clinical psychologists (Austria) or trained evaluators with high school education in the field of social care (Czech Republic). Furthermore, all non-direct tested information (e.g. nutritional status, activities of daily living status, number of falls and hospital stays) was, in Austria, collected by a research assistant, recruited from the respective nursing home care team for the duration of the data collection.

**Table 1:** Overview of data files/data sets [3, 4]

Label	Name of data file/data set	File types (file extension)	Data repository and identifier (DOI or accession number)
Data file 1	DEMDATA_rawdata_reduced	.csv	10.5281/zenodo.2547431
Data file 2	DEMDATA_BMC_Description_variables	.pdf	10.5281/zenodo.2451261

In order to assess the cognitive status, the Global Deterioration Scale (GDS, [5]), the Mini-mental status examination (MMSE, [6]) and the Clock-drawing test ([7]) were used. Concentration, short-term memory, long-term memory and orientation were assessed with the 7-point Brief Cognitive Rating Scale (BCRS, [8]). Behavioral symptoms were rated on the BEHAVE-AD-FW which assesses seven domains of behavioral pathology in Alzheimer’s disease (i.e., delusions, hallucinations, activity disturbances, aggressiveness, diurnal rhythm disturbances, affective disturbances, anxieties and phobias) as reported by a member of the care team [9]. In the current data, the sum score across all subscales is available. In addition, the Empirical Behavioral Pathology in Alzheimer’s Disease Assessment Scale (E-BEHAVE-AD, [10]) was used as a direct observational version of the BEHAVE-AD-FW. It assesses the same categories as the BEHAVE-AD-FW (except diurnal rhythm disturbances) and was filled in based on the direct interview with the resident by the clinical psychologist. Functioning was assessed with the Functional Assessment Staging (FAST, [11]) and the KATZ Index of Independence in Activities of Daily Living [12]. For the KATZ Index, individual Items and the sum score are provided in the data. Mobility was assessed via the Timed-get-up-and-go Test [13]. In order to assess the residents’ quality of life, the Quality of Life in Alzheimer’s disease (QOL-AD; person with dementia-version, [14]) and the Euroquol (EQ) 5D-3L scale [15] were used. As the QOL-AD was administered only in Austria, this variable was removed from the data set in order to guarantee residents’ privacy. The EQ-5D-3L, consists of five questions (agility/mobility, care for oneself, usual activity, pain, anxiety/depression). Furthermore, the resident had to assess his or her health status on a visual analogue scale. Physical pain was assessed (in Austria only and hence excluded from the data) by the VAS-Pain scale [16] and, for both countries, by the Pain Assessment in Advanced Dementia (PAIN-AD, [15]). Finally, the short form of the Mini Nutritional Assessment (MNA) was used to assess the nutritional status [17].

## Limitations

To guarantee anonymity of the study participants, we did not include any sociodemographic data such as age, gender or nationality in the data set. Moreover, as some of the test instruments (i.e., VAS pain scale, QOL-AD) were only performed in Austria but not in the Czech Republic, also the results from these assessments were excluded. However, the full data set including age and gender can be provided upon individual request. Furthermore, despite the common study protocol and the same method of nursing home selection in both countries, there were some differences during the data collection between the two countries. For example, entry criteria to nursing homes are different in Austria and the Czech Republic and some of the wards of the Czech nursing homes were not made accessible which may have caused a bias in the data. Therefore, other studies using a similar methodology should clarify how representative our data are for European nursing homes.

## Data Availability

The data described in this Data note can be freely and openly accessed via https://zenodo.org. Please see Table 1 and Refs. [3, 4] for details and links to the data.

## Abbreviations

BEHAVE-AD-FW: Behavioral Pathology in Alzheimer’s Disease Assessment Scale/Frequency-Weighted
BCRS: Brief Cognitive Rating Scale
E-BEHAVE-AD: Empirical Behavioural Pathology in Alzheimer’s Disease Assessment Scale
Euroquol/EQ-5D-3L: Assessment of Quality of life and health outcome
FAST: Functional Assessment Staging of Alzheimer Disease
GDS: Global Deterioration Scale
KATZ Index: ADL Scale
MMSE: Mini mental state examination
MNA: Mini Nutritional Assessment
PAIN-AD: Pain Assessment in Advanced Dementia-scale
QOL-AD: Quality of Life in Alzheimer’s disease
VAS-pain scale: visual analogue scale pain scale
